# C2 IgM Natural Antibody Enhances Inflammation and Its Use in the Recombinant Single Chain Antibody-Fused Complement Inhibitor C2-Crry to Target Therapeutics to Joints Attenuates Arthritis in Mice

**DOI:** 10.3389/fimmu.2020.575154

**Published:** 2020-10-16

**Authors:** Nirmal K. Banda, Stephen Tomlinson, Robert I. Scheinman, Nhu Ho, Joseline Ramos Ramirez, Gaurav Mehta, Guankui Wang, Vivian Pham Vu, Dmitri Simberg, Liudmila Kulik, V. Michael Holers

**Affiliations:** ^1^Division of Rheumatology, Department of Medicine, University of Colorado Anschutz Medical Campus, Aurora, CO, United States; ^2^Department of Microbiology and Immunology, Medical University of South Carolina, Charleston, SC, United States; ^3^Skaggs School of Pharmacy and Pharmaceutical Sciences, University of Colorado Anschutz Medical Campus, Aurora, CO, United States

**Keywords:** natural antibodies, single chain, C2-Crry, complement, complement inhibitor, arthritis

## Abstract

Natural IgM antibodies (NAbs) have been shown to recognize injury-associated neoepitopes and to initiate pathogenic complement activation. The NAb termed C2 binds to a subset of phospholipids displayed on injured cells, and its role(s) in arthritis, as well as the potential therapeutic benefit of a C2 NAb-derived ScFv-containing protein fused to a complement inhibitor, complement receptor-related y (Crry), on joint inflammation are unknown. Our first objective was to functionally test mAb C2 binding to apoptotic cells from the joint and also evaluate its inflammation enhancing capacity in collagen antibody-induced arthritis (CAIA). The second objective was to generate and test the complement inhibitory capacity of C2-Crry fusion protein in the collagen-induced arthritis (CIA) model. The third objective was to demonstrate *in vivo* targeting of C2-Crry to damaged joints in mice with arthritis. The effect of C2-NAb on CAIA in C57BL/6 mice was examined by inducing a suboptimal disease. The inhibitory effect of C2-Crry in DBA/1J mice with CIA was determined by injecting 2x per week with a single dose of 0.250 mg/mouse. Clinical disease activity (CDA) was examined, and knee joints were fixed for analysis of histopathology, C3 deposition, and macrophage infiltration. In mice with suboptimal CAIA, at day 10 there was a significant (*p* < 0.017) 74% increase in the CDA in mice treated with C2 NAb, compared to mice treated with F632 control NAb. In mice with CIA, at day 35 there was a significant 39% (*p* < 0.042) decrease in the CDA in mice treated with C2-Crry. Total scores for histopathology were also 50% decreased (*p* < 0.0005) in CIA mice treated with C2-Crry. C3 deposition was significantly decreased in the synovium (44%; *p* < 0.026) and on the surface of cartilage (42%; *p* < 0.008) in mice treated with C2-Crry compared with PBS treated CIA mice. Furthermore, C2-Crry specifically bound to apoptotic fibroblast-like synoviocytes *in vitro*, and also localized in the knee joints of arthritic mice as analyzed by *in vivo* imaging. In summary, NAb C2 enhanced arthritis-related injury, and targeted delivery of C2-Crry to inflamed joints demonstrated disease modifying activity in a mouse model of human inflammatory arthritis.

## Introduction

Rheumatoid arthritis (RA) is the leading cause of autoimmune arthritis, and as the population ages RA-related disabilities in patients in the US have been projected to increase over the next 25 years by 40% (1), suggesting that this disease will continue to impact the public health care system dramatically (2). While the pathogenesis of RA is complex and no single mechanism or biomarker can explain its initiation and perpetuation, many potential risk factors for the development of RA have been identified (3). The complement system (CS), a component of innate immunity, plays an important role in the pathogenesis of RA (4). Complement activation is essential for disease progression in active and passive transfer mouse models of RA, and activated complement fragments have been found in the synovium of RA patients (5–7). Specifically, the alternative pathway (AP) and C5aR of complement is required for the perpetuation and severity of disease, as mice lacking complement MASP-1/3, factor D, factor B, C5, and C5aR are substantially resistant to arthritis while mice lacking C1q, C4, mannose-binding lectin, FCN A, Collectin 11, and FCN A are susceptible to arthritis (5, 8–12). In addition, mice lacking C3, C3aR, FCN B, and MASP-2 are partially resistant to arthritis (8, 13). Complement activation products have been shown to be generated locally during the pre-clinical initiation of RA (14). Furthermore, CS-based therapeutics have shown excellent therapeutic efficacy in mouse models of RA (15, 16), but have failed in RA clinical trials due to the complex nature of the late stage disease and potentially a failure to reach the injured joints in a sufficiently high concentration. For this and other reasons, there is an unmet need for the development of new therapeutics based on advancing understanding of the CS and its ability to damage local tissues in sites such as the joint through interactions with injured and apoptotic cells.

The CS gets activated by three different pathways: the classical pathway (CP), the lectin pathway (LP), and the AP (4). All of these pathways generate two potent pro-inflammatory molecules; C3a and C5a, via C3 and C5 convertases, respectively, as well as the C5b-9 membrane attack complex and C3 fragment ligands for other complement receptors. Normally, host cells are protected from an inadvertent self-attack of the CS through immediate intervention by deactivation of the early C3/C5 convertases steps or during the late assembly of the membrane attack complex (17). The deactivation of complement C3/C5 convertases is mediated by several soluble and membrane bound regulatory proteins (17) whose role is to protect tissue and organs from inappropriate CS-mediated damage. Both soluble and membrane bound regulatory proteins are essential to prevent immunopathology resulting from over activation of the CS in various autoimmune diseases such as RA. Specifically in mouse, one such membrane bound regulatory protein known as CR1-related gene/protein Y (Crry), a product of the *Crry* gene, is present. Crry is equivalent in several ways to human membrane cofactor protein (MCP; CD46) and also complement receptor 1 (CR1; CD35). CD46, CR1 and Crry are cofactors for the factor I (FI)-mediated cleavage of C3b and C4b, which are active products of C3 and C4 complement proteins (18). In contrast to MCP, though, Crry also possesses decay-accelerating activity for the CP but weak decay-accelerating activity for the AP C3 convertase (19–21). Crry has been shown to maintain homeostasis in mice, and mice lacking Crry demonstrate an embryonically lethal phenotype (21, 22). Crry has been shown play an important role in autoimmune disease such as lupus (MRL/lpr mice) (23, 24) and Alzheimer’s disease (25, 26). However, Crry-Ig alone was not effective compared with anti-C5 mAb in protecting mice from collagen-induced arthritis (CIA) (27), suggesting its non-availability in the damaged joints or cartilage surface. Nonetheless, inhibition of complement by a fusion protein, CR2-Crry has been shown to reduce development of atherosclerosis (28) and collagen antibody-induced arthritis (CAIA) (29, 30). In an experimental autoimmune encephalomyelitis (EAE)-induced demyelination model in mice it has been shown that using adeno-associated viral (AAV)-mediated, *in vivo*, targeted gene delivery of Crry, there was a selective inhibition of activated C3 at presynaptic boutons protected structural synapses and visual function. The authors have achieved the inhibitory effect on C3 by overexpressing Crry fused to a domain of CR2 that only binds activated C3 in retinal ganglion cells, resulting in abundance of Crry protein in retinogeniculate presynaptic terminals following EAE (31).

Despite the presence of membrane bound regulatory proteins, abnormal complement activation occurs in many clinical conditions such as tissue inflammation, myocardial or intestinal ischemia, blunt trauma, hemorrhagic shock, glomerulonephritis, autoimmune diseases, and after exposure to bacterial toxins (32). In systemic autoimmune diseases such as arthritis and lupus, complement activation might be initiated by circulating immune-complexes (ICs), which are characteristic features of these autoimmune diseases (4, 33).

To protect from viral and bacterial infections, healthy individuals express natural antibodies (NAbs), which are normally produced antibodies, subsets of which can recognize self, and/or foreign antigens. NAbs can cause direct neutralization of bacteria or viruses present in the circulation. NAbs/natural autoimmunity forms a network that serves to protect the organisms from exterior and interior danger, but may also contribute to autoimmune diseases (34). While NAbs are generally of the IgM or IgG3 isotype, nonetheless some IgA and IgG NAbs have also been identified (32). NAbs are typically encoded by germline variable (V) genes without extensive mutations (35). The major source of natural IgM are long lived peritoneal CD5 + B1 cells (36–38). It is not clear whether B1a (CD5C) or B1b (CD5K) B cells produce these NAbs (32, 39, 40). B1 cells are long lived which are positively selected for self-reactivity (34). Nonetheless some NAbs are synthesized by splenic CD5^–^B2 cells (39).

In addition to direct or indirect lysis of pathogens by complement, NAbs have been shown to be involved in the clearance of senescent erythrocytes (41), intracellular debris and infectious agents from the circulation (42). Natural IgM has been shown bind to C1q and activate the complement cascade (43, 44). Furthermore, natural IgM also binds mannose-binding lectin (MBL), which is bound to apoptotic cells (45, 46) and clears ICs. These studies show that NAbs and the CS can act in some situations in concert to maintain homeostasis in an apoptotic microenvironment such as injury or inflammation.

In the recent past, we have cloned and purified one IgM NAb, designated C2, a mAb that recognizes an epitope expressed on a subset of phospholipids (PL), and shown that C2 mAb specifically bind to the intestine (47), brain (48), and heart following ischemia-reperfusion injury (49). Furthermore C2 binding to PL is not fatty acid dependent, as it binds to PL with different fatty acids, either saturated or unsaturated, and the epitope is not oxidized on double bound fatty acid (Kulik, L et al. unpublished data). We have also previously shown that certain IgM NAbs function as innate immune sensors of injury via recognition of neoepitopes expressed on damaged cells or within the spinal cord after injury (50). Other studies have shown that only cells undergoing apoptosis, but not normal viable cells, generate oxidation-specific neoepitopes, including biologically active oxidized low density lipoproteins, which in turn serve as dominant autoantigens as well as provide “pro-inflammatory” signals, mediating autoimmune and inflammatory responses (51).

In addition to tissue injury, many chemotherapeutics or biologics ultimately induce apoptosis as their mode of action (52, 53). Therefore, from a drug development prospective C2 as a mAb directed against specific apoptosis-induced PL biomarkers can be utilized for the targeted delivery of a complement inhibitory protein, for example, Crry, to create the tissue-targeted fusions protein C2-Crry. We hypothesized that a C3 complement inhibitor protein such as Crry can be directly and specifically delivered via C2 NAb targeting to the injured site, i.e., in the joints of arthritic mice, through binding of inflammation-associated PL generated during injury or inflammation. To this end, based on C2 neoepitope identification during injury, we constructed an anti-C2 single chain antibody (scFv), and then C2 scFv was linked to Crry to create a new fusion protein, C2-Crry with 6Histag. ScFv comprise the variable regions of the heavy (V_H_) and light chains (V_L_) of immunoglobulins, connected with a short linker peptide of ten to about 25 amino acids. We also hypothesized that these C2-specific neoepitopes are generated in the synovium or on the cartilage surface during inflammation in human RA and, therefore, Crry can be delivered clinically as the C2-Crry fusion protein to the site of inflammation, i.e., in the joints in early or late RA. Herein, to provide the proof-of-concept, we have used two mouse models of human RA, anti-collagen antibody induced arthritis (CAIA) and CIA. First to show that C2 mAb by itself, in mice, can enhance injury, i.e., arthritis, we used the mouse model of CAIA, which is dependent on the CS for it is involved in the effector phase of the arthritis. Mice deficient in C3 developed less severe arthritis while mice deficient in C5 are resistance of arthritis (54, 55). Second, to determine if C2-Crry as a fusion protein inhibitor can attenuate injury via suppression of the CS in the joints of mice, we used CIA, which is dependent on T and B cells, although complement is involved in the effector phase in this model. In CIA, active immunization with bovine type II collagen leads to the development of arthritis in which T and B cells are present, but not when preformed antibodies or T cells are used for disease induction (56). We also provided evidence using imaging analysis that Infrared Dye 800 (IRDye 800) labeled C2-Crry localized specifically within the damaged joints of arthritic mice.

## Materials and Methods

### Cloning of C2-IgM NAb Sequences

The variable portion amino acid sequences from the heavy and light chains of C2-IgM with a spacer sequences (4GS)_2_-4G and Crry protein on the C-terminus were cloned into pEE12.4 vector according to published methods (49). The construct was made both with and without 6xHis tag. The protein was produced in stably transfected Chinese Hamster Ovary (CHO) cells.

### Generation and Purification of Natural Antibodies C2 and F632 IgM mAbs

The NAbs C2 and F632 were generated as mAbs and purified according to our previous published study (47). Briefly, C2 and F632 were developed by the fusion of spleen cells from wild type (WT) C57BL/6 mice with the Sp2/0-Ag14 myeloma cell line by the standard protocol to establish hybridomas. To purify C2 and F632 IgM NAbs, exhausted supernatants of cultured C2 and F632 hybridomas, were affinity purified on a column of agarose beads with goat anti-mouse IgM (Sigma-Aldrich, MO, United States). Bound Abs were eluted with a buffer containing 0.1 M glycine (pH 2.3) and collected into a buffer containing 1.5 M Tris (pH 8.8). Eluted mAb were dialyzed against 1 × PBS (pH 7.4) for 48 h and concentrated using centrifugal filtration on Centricon Plus-20 (Millipore, MA, United States). C2 mAb sequences were used to generate the fusion protein, C2-Crry, which recognizes a subset of PL (47), and F632 IgM mAb used as a control recognizing 4-hydroxy-3-nitrophenylacetyl (49). Purified C2 and F632 IgM NAb concentrations for follow up *in vivo* studies were determined by measuring the *A*_280 *nm*_ of the sample, and purity was confirmed by analysis on a 10% SDS-PAGE gel (47).

### Binding of C2 IgM NAb to Apoptotic Thymocytes

Thymocytes were isolated from 6-week old male C57BL6 mice. 6 × 10^6^/ml cells were cultured in DMEM media for 16 h with 1 μM dexamethasone. Each sample of 1 × 10^6^ thymocytes were re-suspended in staining buffer (Ca2+, Mg+ Dulbecco’s Phosphate-Buffered Saline supplemented with 2% Fetal Bovine serum and 0.09% Na_3_N) containing monoclonal antibody for 30 min on ice. Bound C2 antibody was detected with FITC labeled goat anti-IgM FITC. After washing cells were re-suspended in the Flow cytometry (FACS) staining buffer and analyzed. Before running samples through flow cytometer, 1 mg/ml propidium iodide (PI) was added to determine the level of apoptosis in thymocytes. Anti-D5 IgM Nab, which does not bind to PL and has no effect on injury (50), was used as a negative control.

### C2 NAb-Mediated Injury in Collagen Antibody-Induced Arthritis

To examine the effect of purified C2 IgM NAb on arthritis, CAIA, was used as a mouse model of human RA. Wild type (WT) mice on C57BL6 (H-2^*b*^ haplotype) background were used for CAIA (5, 27). A suboptimal level of arthritis was induced in mice by an intraperitoneal (i.p.) injection at day 0 of a single dose (0.5 mg/mouse) of anti-collagen antibody a.k.a. Arthrogen-CIA 5-clone mixture (Chondrex, Inc., Redmond, WA, United States). At day 3, all mice were injected IP with a single dose (50 μg/mouse) of lipopolysaccharide (LPS; Escherichia coli strain; Chondrex, Inc., Redmond, WA, United States). All mice start showing signs of clinical disease, at day 4 when injected with Arthrogen alone but an injection of LPS is required as part of the standard protocol to inducing a sever disease to examine the exact efficacy of test fusion proteins *in vivo*. It has been shown that LPS exacerbate arthritis by inducing the secretion of IL-1β and TNF-α, which are not only involved in immune responses but also in inflammation itself (57). At day 4, i.e., after 24 h of LPS injection mice show the signs of disease and later on start developing severe disease. To determine the effect of anti-C2 NAb, mice were injected three times [0.1 mg/mouse intravenously (i.v.)] with C2 IgM or F632 (*n* = 5) or 1 × PBS (*n* = 5) at day 0, day 3, and at day 5. NAb F632 was used as an internal control to identify the specific pathogenic effects of C2 IgM on arthritis and serves as a Nab control. All mice were sacrificed at day 10. Clinical disease activity (CDA) starting at day 4 was scored each day blindly by two trained laboratory personals according to our published methods (5, 27).

### Cloning, Expression and Purification of the C2-Crry Fusion Protein

The pEE12.4/C2scFv-Crry plasmid was transfected into Expi293 cells, which were grown for 7 days, following which the supernatants were collected and loaded on the His60 Ni Superflow column to purify the proteins. The pEE12.4/C2scFv-Crry plasmid encodes the six His tag at the C terminus of the peptide so protein was bound and then purified from Nickle column (His60 Ni Superflow Resion; Clontech company). The plasmids were transient transfected into Expi293 cells (Expi293^TM^ Expression System Kit, https://www.thermofisher.com/order/catalog/product/A14635) for 7 days, and the supernatant were collected and loaded on the His60 Ni Superflow column for C2-Crry purification (50).

### Flow Cytometry Analysis for Binding of C2-Crry to Apoptotic Human Umbilical Vein Endothelial Cells

To determine whether the purified C2-Crry fusion protein binds to apoptotic human umbilical vein endothelial cells (HUVECs), flow cytometry was used. HUVEC cells were incubated over night with 10 mM Antimycin A (AMA) to induce apoptosis. Cell were de-attached from plates using Accutase (Sigma). Cells were incubated on ice for 30 min with C2-Crry protein at 10 μg/ml. The bound protein was detected with rabbit anti-6xHis-FITC. Flow cytometry analyses was performed on cells gated for live (G1 gate) and apoptotic (G2 gate) cells.

### Binding of C2-Crry to the Phospholipid, Cardiolipin

Enzyme-linked immunosorbent assay (ELISA) was used to determine whether the purified C2-Crry fusion protein binds to specific PL. The ELISA plates were pre-coated over night with 10 μg/ml of cardiolipin (CL; phospholipid) re-suspended in Methanol and stored at 4°C with ELISA wells left uncovered for Methanol to evaporate overnight. PC-BSA (Phosphorylcholine hapten conjugated to bovine serum albumin by use of p-diazonium phenylphosphorylcholine) was added to plates in amount 5 μg/ml in PBS. After blocking the ELISA plates with BSA, different concentrations of C2-Crry were applied to wells. Bound C2-Crry was detected with anti-6xHis antibody.

### Effect of C2-Crry on Collagen-Induced Arthritis

To examine the direct effect of C2-Crry fusion protein on disease, CIA, another mouse model human RA, was evaluated. Wild type DBA 1/J (H-2^*q*^ haplotype) mice were used due to the high susceptibility of this strain to CIA. CIA in mice was induced at day 0 by injecting an emulsion intra-dermally at the base of the tail containing a mixture of bovine type II collagen (BCII; 200 μg/mouse; Sigma) plus *Mybacterium tuberculosis* (MT; 200 μg/mouse; Complete Freund’s Adjuvant; CFA; H37Ra; Difco Laboratories, Detroit, MI, United States). A booster injection was given at day 21 using identical doses of BCII and MT (58). To determine the inhibitory effect of C2-Crry, mice with CIA (*n* = 14), were injected with a dose of 0.250 mg/mouse/i.p. or 1 × PBS control 2x per week after the booster injection of CII/CFA at day 21. All mice were sacrificed at day 35. CDA was examined according to our published methods (58). At day 35, all four limbs (two forepaws, two hind paws with knee joint and ankle) were surgically removed and fixed in 10% neutral buffered formalin (NBF) for histopathological analysis (58).

### Histopathology and Immunohistochemical Staining for C3 Deposition and Macrophage Determination

All four joints (both fore limbs and the right hind limb, with knee joint, ankle and paw) from DBA 1/J mice with CIA treated with C2-Crry or controls and sacrificed at day 35 were fixed in 10% NBF to examine for overall histopathological changes and C3 deposition in the synovium and on the surface of cartilage. From CAIA mice, no limbs were processed for histopathology and C3 deposition. Histopathology with scores for inflammation, pannus, cartilage damage and bone damage was assessed by using Toluidine-blue (T-blue) according to our published criteria (5, 10, 15, 27, 58). C3 deposition was assessed by using a primary polyclonal goat anti-mouse C3 Ab (dilution 1:10,000; ICN Pharmaceuticals, Costa Mesa, CA, United States) and detected by goat anti-HRP polymer kit per manufacturer’s instructions (Biocare Medical, Concord, CA, United States). immunohistochemical (IHC) visualization of C3 protein was carried out using 3′, 3′diaminobenzidine solution substrate (DakoCytomation, Carpinteria, CA, United States) that reacts with HRP and generate a brown color stain on the sections. The presence of macrophages in the knee joints was also quantitated by IHC using F4/80 according to our published methods (13).

### Measurements of Anti-collagen Antibodies

Serum was collected from each mouse with CIA treated with C2-Crry or controls by retro-orbital bleeding under anesthesia on day 0 (right eye), on day 21 (left eye), and on day 35 (right eye). Samples were used in triplicate to measure IgG1 and IgG2a anti-mouse CII antibodies by ELISA at a dilution of 1:2000 in 1 × PBS as previously describe (27, 58). A standard pool of anti-CII antibodies was made by mixing sera from several mice with severe arthritis and used to create a standard curve. Absorbance was read at 405 nm with a 492 nm correction filter.

### Fibroblast-Like Synoviocyte Culture

Primary fibroblast-like synoviocytes (FLS) derived from mice with CIA (originally obtained from Dr. Gary Firestein (University of California–San Diego) were cultured in T25 tissue culture flasks in DMEM high-glucose medium (Sigma) containing 10% FBS, 1% penicillin-streptomycin, 1% L-glutamine, and 0.5% gentamicin (59). The FLS became confluent at 7–14 days. Cultured FLS were used up to 10 passages when a new aliquot of cells was thawed. FLS were used for evaluation of binding of IRDye 800 labeled C2-Crry to the apoptotic FLS.

### Induction of Apoptosis in FLS

Apoptosis in FLS 1 × 10^5^ in 0.5 ml media in a 24-well plate were induced by using serum starvation for hours 72 h to assess the binding of C2-Crry to apoptotic FLS. Serum was withdrawn from the FLS culture for 72 h to trigger apoptosis. Apoptosis in FLS was also confirmed using 1.2% agarose gel electrophoresis and identification of a cleaved DNA ladder.

### Labeling of C2-Crry With IRDye 800CW

To show the binding of C2-Crry *in vitro* to apoptotic FLS, we labeled the protein with IRDye800-NHS ester (Li-COR Biosciences) as we have shown previously (60; 61). C2-Crry fusion protein was incubated with a 10-fold molar excess of dye for 5 h at 4°C in PBS. Unbound dye was removed with a 7 kDa Zeba desalting column (Thermo Fisher). After labeling C2-Crry with IRDye800 and addition to a Zeba column, the protein was eluted from the column with PBS and stored at 4°C before use according to our previously published study (60; 61). Similarly, Arthrogen (anti-CII) mAbs were labeled with IRDye 800 (60; 61).

### Near-Infrared Microscopy for *ex vivo* IRDye 800 Labeled C2-Crry Binding With Apoptotic FLS

IRDye 800CW labeled C2-Crry (10 μg/ml) was added to the serum starved apoptotic FLS in a 24-well plate and incubated for 2 h at room temperature. Serum starved FLS for 6 h were used as negative and positive controls to show specific FLS apoptosis followed staining with Caspase 3/7 (2 mM) green detection reagent using a glass slide according to manufacturer’s standard protocol (ThermoFisher Scientific). The cells were fixed in 4% formalin and imaged with Zeiss Axio Observer 5 epifluorescent microscope equipped with X-Cite 200DC light source and Axiocam 506 monochromatic camera. Near infrared fluorescence was detected using a Cy7 filter set, catalog number 49007, Chroma Corporation (McHenry, IL, United States). The plate fluorescence was read with a Li-COR Odyssey scanner at 800 nm, and the integrated density of gray 8-bit images (TIFF) of the wells was determined with ImageJ software.

### *In vivo* Joint-Specific Detection of IRDye 800 Labeled Anti-CII Abs and C2-Crry

To examine joint-specific distribution of IRDye 800 labeled C2-Crry in WT C57B6 mice with CAIA, two experiments were done. First, to show that IRDye 800 labeled anti-CII Abs alone binds to the cartilage in the joints, and dye itself have no effect on antibody binding capacity, C57B6 WT mice were injected with a single low dose of IRDye 800 labeled anti-CII Abs (Arthrogen; 50 μg/mouse/i.v.) or murine IgG (50 μg/mouse/i.v.) or non-injected. After two weeks of antibody wash out period (15 days) mice were sacrificed to detect IRDye 800 labeled anti-CII Abs in the joints. IRDye 800 labeled anti-CII Abs clears very slowly from the circulation but firmly binds to the cartilage in joints even after a long wash period according to our published methods (60; 61). This binding of IRDye 800 anti-CII Abs also induced a low level of disease and it can compete with the unlabeled arthrogen (60). Our previous study show that IRDye 800 labeled anti-CII also does not bind non-specifically to spleen, kidney, lung, heart, liver and intestine but only to the arthritic joints (61). At day 15, bones of forelimbs and hind limbs were cleaned of muscle, sandwiched between two glass slides and scanned with a Li-COR Odyssey at 700 nm channel for autofluorescence and 800 nm channel for IRDye 800. Mean fluorescence was determined from 8-bit images using ImageJ. No gross morphology and bright field images were taken due to clarity issues.

Second to show the specific binding of the IRDye 800 labeled fusion protein C2-Crry to the damaged cartilage or to the joints, a single dose of C2-Crry (50 μg/mouse/i.v.) was injected at day 15 in mice with suboptimal disease, i.e., CAIA. A suboptimal or a low level of disease was induced by using 50 μg/mouse of unlabeled Arthrogen i.v. followed by LPS (50 μg/mouse/i.p.) which is sufficient to bind and cause mild injury in the joints as mentioned above. At day 18, IRDye 800 labeled C2-Crry injected (i.v.) mice were sacrificed and scanned using Li-COR Odyssey as mentioned above. For control, mice were injected with unlabeled C2-Crry or not injected with anything. In order to determine the circulation half-life of IRDye 800 labeled C2-Crry, plasma collected at different time points during the 3 day (day 15 to day 18) period was applied in 2 μl triplicates on a 0.22 μm nitrocellulose membrane and scanned at 800 nm using Li-COR Odyssey (60). For this study, no gross morphology and bright field images were taken.

### Statistics

*p*-values were calculated using Student’s *t* test with the GraphPad Prism^®^ 4 statistical program. ANOVA was used for CDA, histopathology, C3 deposition and macrophage data. The data in graphs, histograms and tables are shown as the mean ±SEM, with *p* < 0.05 considered significant.

## Results

### Flow Cytometry Analysis Showing Binding of C2 IgM NAb to Apoptotic Thymocytes

**FIGURE 1:**
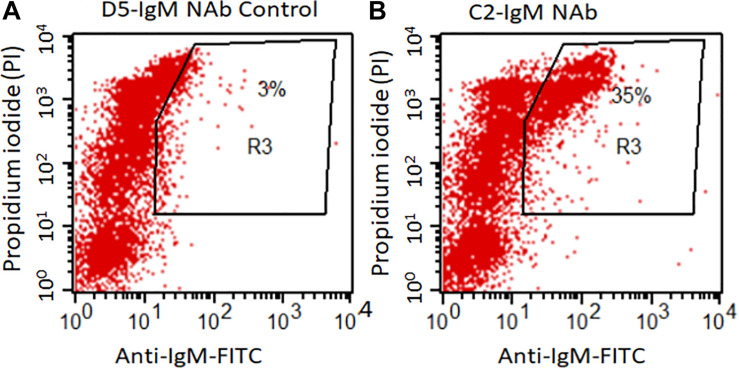
C2-IgM antibody recognizes a subpopulation of late apoptotic thymocytes. Mouse thymocytes were cultured for 16 h in the presence of 1 mM Dexamethasone; apoptotic thymocytes were then analyzed by FACS analysis for the binding of C2-IgM antibody. **(A)** FACS analysis showing minimal (background level) binding of a non-specific NAb, anti-D5 IgM (anti-cytokeratin 19 antibody) to apoptotic cells. **(B)** Anti-C2-IgM NAb specific binding was detected on a subset of late apoptotic cells (PI^*high*^).

Flow cytometry data show a significantly higher levels of anti-C2-IgM NAb binding to late apoptotic mouse thymocytes compared to control anti-D5-IgM NAb binding (Figure 1). At 16 h, 35% of dexamethasone treated thymocytes were positive for anti-C2-IgM antibody compared with only 3% for control anti-D5-IgM antibody (Figures 1A,B). Thus NAb C2-IgM specifically binds to apoptotic cells, rationalizing its study for enhancement of arthritis or its incorporation as a component of an scFv-linked complement inhibitor to deliver complement inhibition activity to injured cells or tissues.

### Enhancement of Arthritis by C2-IgM NAb

The CAIA mouse model, wherein damage is dependent on an intact CS, was used to examine the potential inflammation or injury enhancing effect of C2-IgM NAb in suboptimally induced arthritis.

**FIGURE 2:**
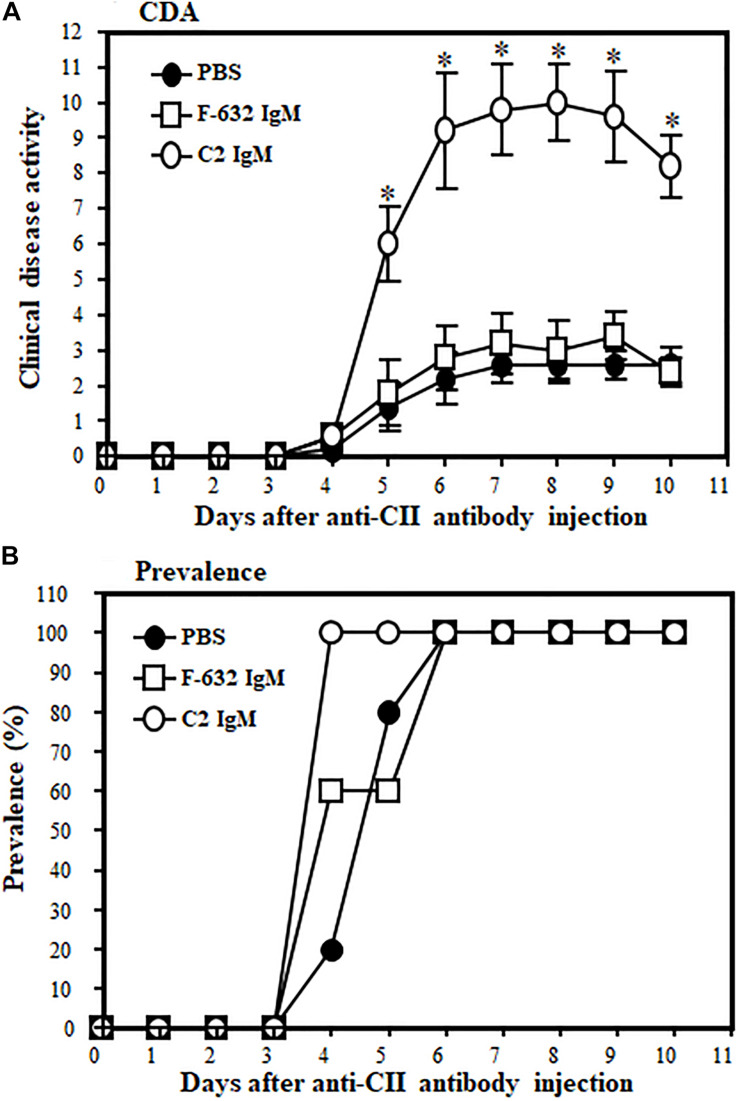
Showing the disease modifying effect of C2-IgM or F632-IgM NAbs on suboptimal CAIA. A suboptimal level of arthritis in mice was induced by injecting Arthrogen 4 clone (anti-CII abs; 0.5 mg/mice) and LPS as mentioned in the Materials and Methods. Mice were injected (i.v.) with a single dose (0.1 mg/mouse) of C2-IgM (*n* = 5) or F632-IgM (*n* = 5) or 1 × PBS buffer control (*n* = 5) at day 0 (prior to arthritis induction with anti-CII abs), day 3 (prior to LPS injection), and at day 5 (after the induction of arthritis). Mice injected with F632-IgM or 1 × PBS served as controls. **(A)** An increase in the CDA in mice injected with C2-IgM compared with F632-IgM. **(B)** Prevalence (%) of disease. Data shown as Mean ± SEM. *p < 0.05 considered significant.

All WT mice were injected i.v., three times, before, during and after induction of disease, either with 1 × PBS or F632 IgM or C2 IgM (Figure 2). At day 10, the CDA was 2.6 ± 0.509, 2.4 ± 0.400 and 9.2 ± 0.374 in mice treated with 1 × PBS, F-632 Nab, and C2-IgM Nab, respectively (Figure 2A). Overall, there was a 74% (*p* < 0.017) and 72% significant (*p* < 0. 006) increase in the CDA in mice treated with C2-IgM compared with mice treated with control F632 NAb or 1 × PBS, respectively. At day 10, the prevalence of disease was 100% in all groups (Figure 2B). There was no toxic effect on mice for all experimental mice survived and also there was no significant loss of weight. These CDA data show that anti-C2-IgM NAb enhanced suboptimal inflammation to generate severe near maximal inflammation in CAIA mice. Representative pictures of forelimbs of mice with visible enhancement of inflammation or injury by C2-IgM NAb are shown in Supplementary Figure 1.

### Purification and Assessment of the Quality of C2-Crry Fusion Protein

**FIGURE 3:**
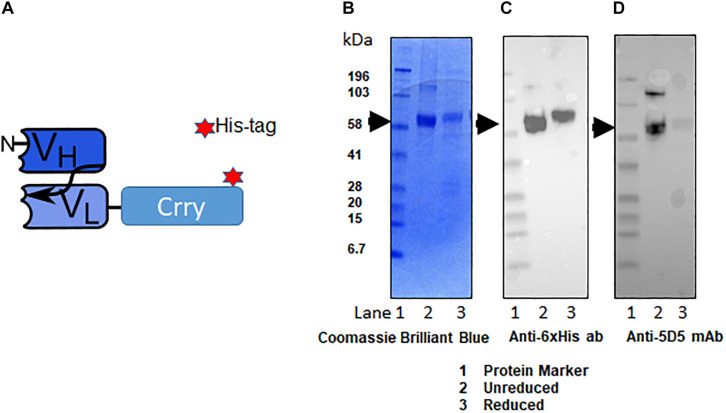
Schematic representation of scFv-Crry construct and purification of C2-Crry fusion protein. **(A)** The variable portion amino acid sequences from heavy chain of C2-IgM and light chain C2-IgM with a spacer sequences (4GS)_2_-4G and Crry protein on the C-terminus were cloned into pEE12.4 vector. The construct was made either with 6xHis tag or without 6xHis tag. The His tagged and non-His tag C2-Crry were purified from CHO cell supernatant by affinity chromatography on bound to sepharose anti-Crry mAb 5D5. Purified C2-Crry protein either was loaded into the gel in the loading sample buffer (1-unreduced) or boiled in a loading sample buffer containing 2-mercaptpethanol as a reducing agent (2-reduced). **(B)** The identity of purified C2-Crry band was confirmed separating reduced or unreduced samples using 10% Tris–glycine SDS gel followed by staining with Coomassie Brilliant Blue. **(C)** Western blot analysis confirming C2-Crry protein band (65.5 kDa) using HRP-conjugated anti 6xHis tag mAb. **(D)** Western blots using anti-Crry antibody, 5D5 confirmed the presence of Crry along with C2 in the C2-Crry fusion protein.

The fusion protein C2-Crry was originally generated tagged with a hexa-histidine tag (6xHis-tag; Figure 3A). C2-Crry was purified and its size of 65.5 kDa was confirmed by SDS-PAGE (Figure 3B lanes 1, 2). Commercially available anti-His-tag antibody was used to check the quality of C2-Crry fusion protein (Figure 3C lanes 1, 2). In parallel, additional Western blot confirmed that anti-Crry mAb 5D5 binds to a Crry conformational epitope, as reduced C2-Crry does not reveal any 5D5 epitope (Figure 3D, lanes 1, 2). Since 5D5 binds to Crry only, Western blot confirmed the presence of Crry in the C2-Crry fusion protein (Figure 3D, lanes 1, 2).

### Binding of C2-Crry Fusion Protein to Apoptotic HUVECs

**FIGURE 4:**
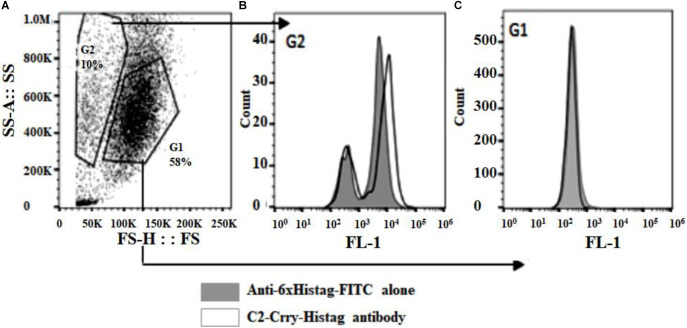
Purified C2-Crry protein was analyzed by flow cytometry and demonstrated to bind apoptotic HUVEC cells. The HUVEC cells were incubated over night with 10 μM Antimycin A to induce apoptosis. After harvesting, cells were incubated on ice for 30 min with C2-Crry protein (10 μg/ml). The bound C2-Crry protein was detected with rabbit anti-6xHis-FITC conjugated antibody. **(A)** Flow cytometry analyses was performed on cells, **(B)** on apoptotic cells (boxed in G2 gate), or **(C)** on healthy cells (boxed in G1 gate).

To determine if the C2-Crry fusion protein binds specifically to apoptotic HUVEC cells, apoptosis was induced using AMA as described in Materials and Methods. FACS analysis confirmed that C2-Crry preferentially binds to apoptotic cells present in the G2 gate as compared to the G1 gate. Bound C2-Crry protein was detected with rabbit anti-6xHis –FITC (Figures 4A–C).

### Enzyme-Linked Immunosorbent Assay Showing Binding of C2-Crry to Phospholipids

**FIGURE 5:**
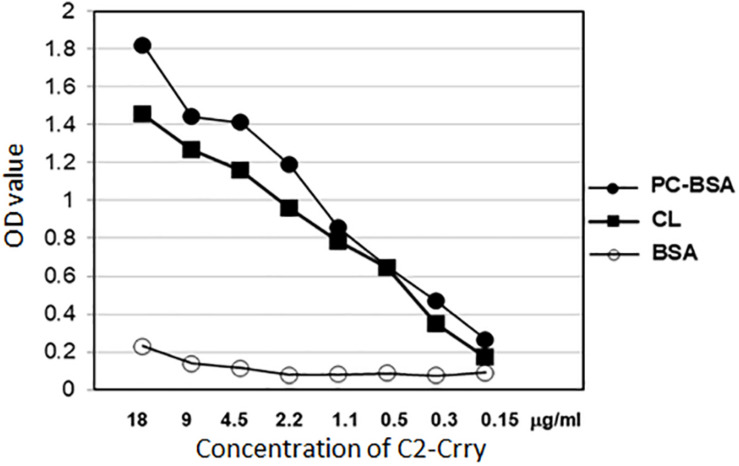
Purified C2-Crry protein was analyzed in ELISA for binding the phospholipids cardiolipin (CL) and phosphatidylcholine-*b*ovine serum albumin (PC-BSA). The ELISA plates were covered over night with 10 μg/ml of cardiolipin dissolved in methanol and stored at 4°C with ELISA wells uncovered for methanol to dry out through the night. PC-BSA was added to plates in amount 5 μg/ml in PBS. After blocking the plates with BSA, different concentrations of C2-Crry (*X*-axis) were applied to wells. BSA was used as a control. Bound C2-Crry was detected with anti-6xHis antibody. Data shown as raw mean OD values (*Y*-axis). All samples were run in duplicate.

Enzyme-linked immunosorbent assay was used to demonstrate retention of the specificity of C2-Crry binding to PL of the class present on the surface of apoptotic cells. ELISA results show that C2-Crry has similar PL characteristics as reported for C2 IgM (Figure 5). C2-Crry fusion protein specifically bound to CL and Phosphorylcholine-Bovine Serum Albumin (PC-BSA) in a dose dependent manner, but did not bind BSA alone (Figure 5). CL is a unique PL which is located in the inner mitochondrial cell membrane, and was specifically recognized by C2-Crry.

Phosphorylcholine-Bovine Serum Albumin was also used (Figure 5). In PC-BSA, Phosphorylcholine hapten is conjugated to BSA protein by use of p-diazonium phenylphosphorylcholine. Our ELISA data (Figure 5) and above FACS data (Figure 4) confirm that C2-Crry can specifically recognize PL found on the surface cells undergoing apoptosis.

### Amelioration of Arthritis by Treatment With the C2-Crry Fusion Protein

**FIGURE 6:**
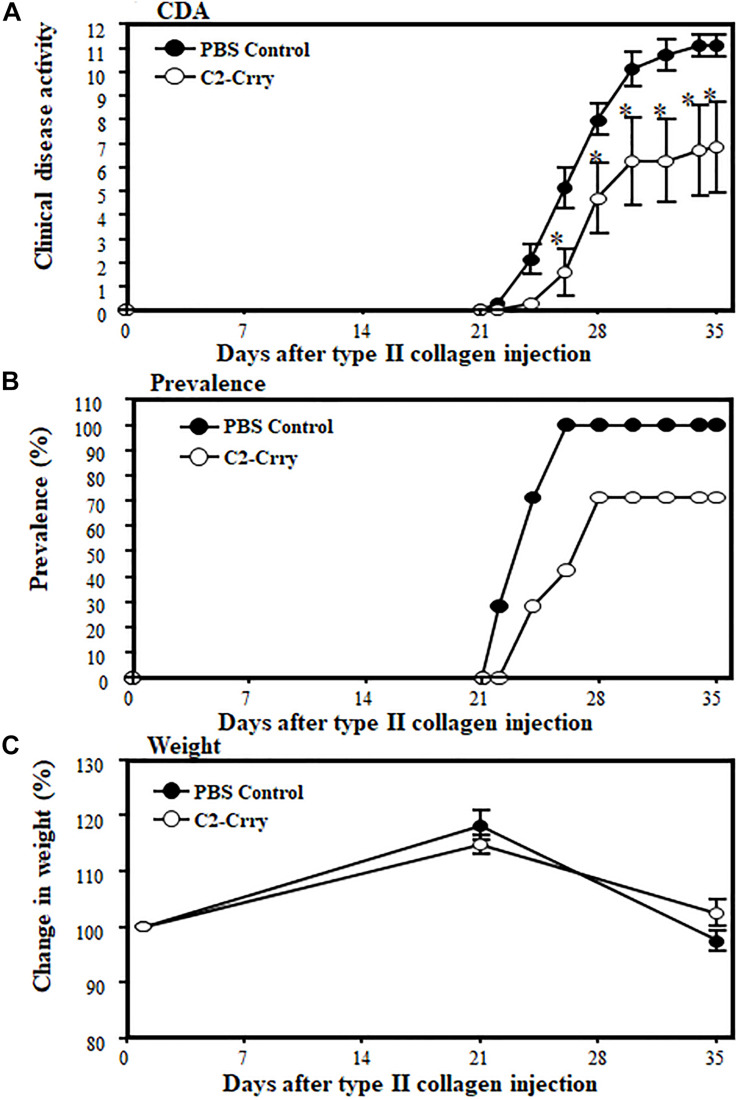
Inhibitory effect of C2-Crry on the development of CIA. Mice were injected 4 times with a dose of 250 μg/mouse/i.p. of C2-Crry (*n* = 7) or 1 × PBS (*n* = 7) control 2x per week after the booster injection of type II collagen/CFA at day 21, at day 25, at day 29, and at day 32 as described in the Materials and Methods. All mice were sacrificed at day 35. **(A)** Mice injected with C2-Crry were protected (39%) significantly compared with mice treated with 1 × PBS. **(B)** Prevalence (%) was 66% and 100% in mice treated with C2-Crry and PBS, respectively. **(C)** Change in weight (%). Data shown as Mean ± SEM. *p < 0.05 considered significant.

To examine the direct effect of C2-Crry fusion protein on arthritis, we used the CIA model. Mice were injected four times (2 times per week) after the booster injection with a dose of 250 μg/mouse and sacrificed at day 35 (Figure 6). At day 35, the CDA in mice treated with 1 × PBS or C2-Crry was 11.14 ± 0.459 and 6.85 ± 1.89, respectively (Figure 6A). There was a significant 38.5% (*p* < 0.042) decrease in CDA in mice treated with the C2-Crry fusion protein (Figure 6A). The prevalence in mice treated with 1 × PBS or C2-Crry was 100% and 71%, respectively (Figure 6B). There was no significant change in weight in mice treated with C2-Crry compared with 1 × PBS treated mice (Figure 6C). None of the mice showed any type of toxicity, and all mice survived. These CDA and prevalence data in mice with CIA show that C2-Crry specifically decreased arthritis in mice with no toxicity or change in weight of treated mice.

### Decreased IgG2a Anti-CII Levels in CIA Mice Treated With C2-Crry

**FIGURE 7:**
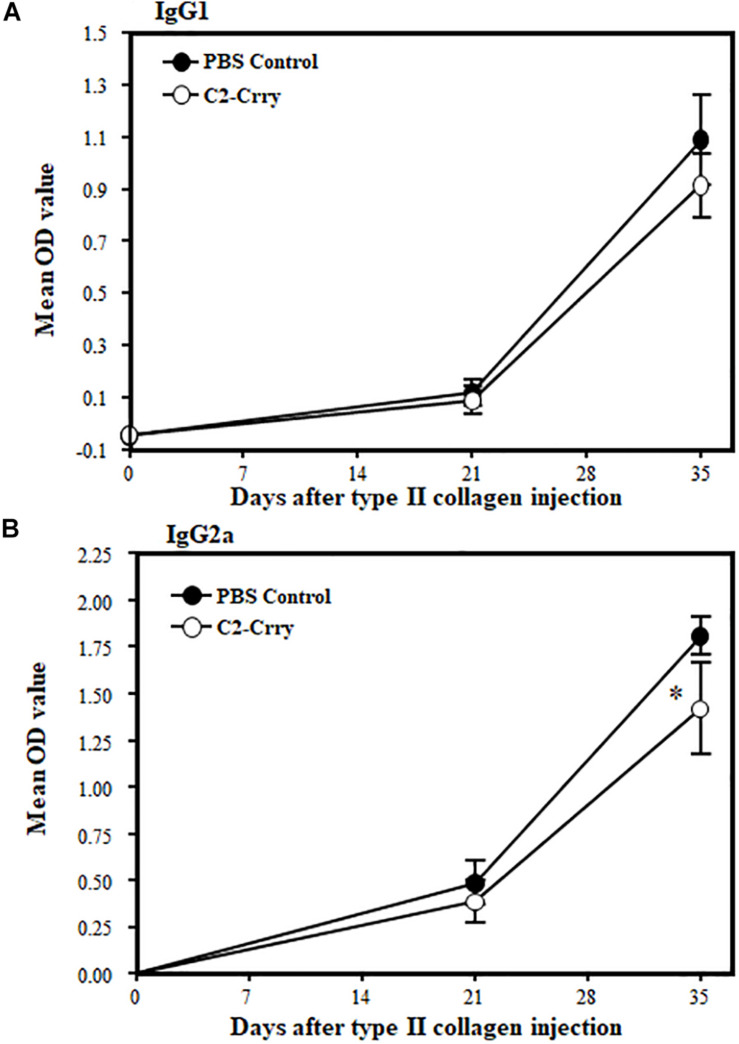
Levels of IgG1 and IgG2a anti-CII antibodies in the sera from mice with CIA after treatment with C2-Crry and PBS. IgG1 and IgG2a antibodies were measured in the serum at day 0 (before induction of disease), day 22 (after booster injection and after one treatment), and at day 35 (after development of disease and after two treatments). **(A)** No decrease in IgG1 in mice treated with C2-Crry (*n* = 7) compared to 1 × PBS (*n* = 7) at day 22 or at day 35. **(B)** Significant decrease in IgG2a in serum in mice treated with C2-Crry (*n* = 7) compared with 1 × PBS control at day 35 only. Data shown as Mean ± SEM. *p < 0.05 considered significant.

IgG1 and Ig2a anti-CII Ab levels in sera from CIA mice treated with 1 × PBS or C2-Crry were examined by ELISA. There was no effect on IgG1 levels (Figure 7A). There was, however, a modest but significant (*p* < 0.048) decrease in the IgG2a anti-CII Ab levels at day 35 compared with PBS treated mice (Figure 7B). These data show that C2-Crry affects pathogenic IgG2a autoantibody levels, which is consist with the decrease in the CDA (Figure 6A).

### Decreased Immunopathology, C3 Deposition but Not in Macrophage Scores in CIA Mice Treated With C2-Crry

**FIGURE 8:**
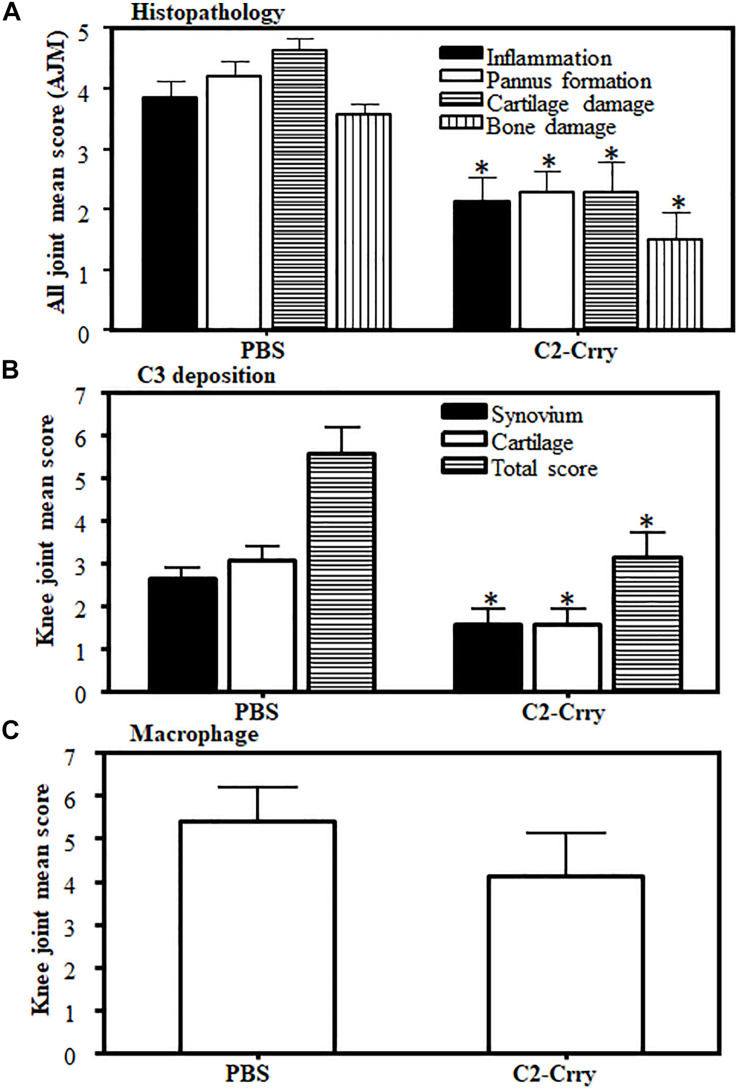
Histopathology scores in CIA mice treated with C2-Crry substantially decreased compared with mice treated with PBS. All joints from mice sacrificed at day 35 were fixed in 10% neutral buffered formalin and processed for histopathology using T-blue and evaluated to determine all joints mean score (AJM). All sections from the joints were cut at a thickness of 5 μm and stained with T-blue. **(A)** Joint sections were scored for inflammation, pannus, cartilage damage and bone damage using a scale of 0–5 for each parameter. Data from all mice were included. **(B)** C3 deposition in the joints of mice treated with C2-Crry compared with 1 × PBS. Mean C3 deposition scores from both the knee joints only in the synovium, on the surface of cartilage and total scores (synovium plus cartilage). **(C)** Macrophage infiltration from the knee joints of mice treated with C2-Crry compared with 1 × PBS. Both the knee joints from each mouse were used to detect the presence of macrophages. All data represent the Mean + SEM comparing C2-Crry (*n* = 7) or with PBS (*n* = 7). *p*-values were calculated using *t*-test. *p < 0.05 in comparison to the mice injected with PBS.

There was a significant (*p* < 0.0005) decrease in immunopathology scores in mice treated with C2-Crry. Immunopathology scores decreased to 44, 46, 51, and 58% for inflammation, pannus formation, cartilage and bone damage, respectively (Figure 8A). C3 deposition was also significantly (*p* < 0.026) 42% decreased both on the cartilage and in the synovium (Figure 8B). No significant decrease in macrophage infiltration was seen in CIA mice treated with C2-Crry compared with the PBS treated mice (Figure 8C). Overall, these immunopathologic and C3 deposition IHC data were consistent with the decrease in CDA seen in mice with CIA (Figure 6A). Representative pictures of T-blue, C3 deposition and macrophage IHC are shown in Supplementary Figure 2.

### *Ex vivo* Targeting of Apoptotic FLS With IRDye 800 Labeled C2-Crry

**FIGURE 9:**
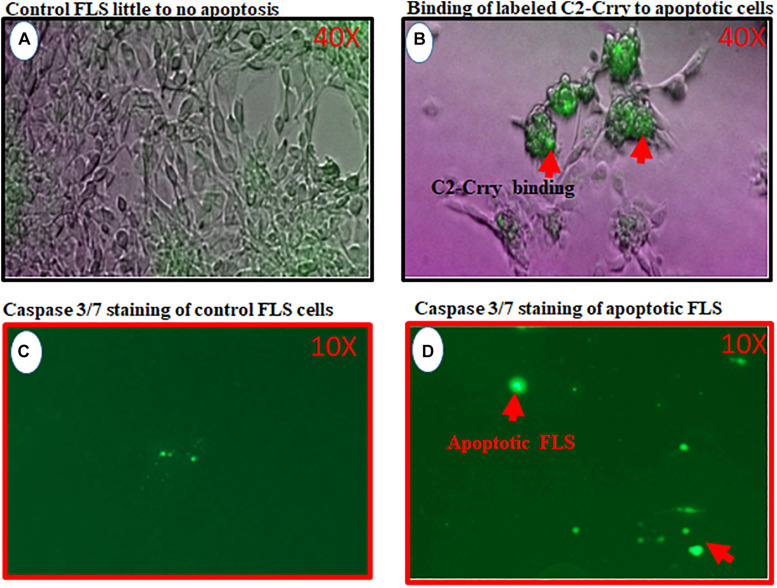
*In vitro* study showing binding of IRDye 800 labeled C2-Crry (Green color) to serum starved FLS undergoing apoptosis. FLS were serum starved in 24-well tissue culture plates for 72 h followed by the addition of IRDye 800 labeled C2-Crry. **(A)** FLS control bright field showing little to no apoptosis. **(B)** Apoptotic FLS bound to labeled C2-Crry (Green color; red arrow) in 24-well plate. **(C)** Caspase 3/7 staining (2 mM) staining used for negative control FLS on the glass slide showing minimum background green color staining. **(D)** Caspase 3/7 staining used to show serum starved apoptotic FLS for 6 h on a glass slide with cover slip. Apoptotic cells and apoptotic bodies appeared green under UV light as shown by red arrows. Scanning/and or imaging using with Zeiss Axio Observer 5 epifluorescent microscope equipped with X-Cite 200 DC light source and Axiocam 506 monochromatic camera. Near infrared Fluorescence was imaged using Cy7 filter set (Chroma Corporation, McHenry, IL, United States). One representative experiment of C2-Crry binding to apoptotic FLS images is shown. No scale was added when the images were taken. But 40x magnification objective was used to take these pictures for images **(A,C)** and 10x objective was used for images **(C,D)**.

To determine that C2-Crry fusion protein target injured synovium in the joints of mice, we used FLS cells derived from the synovium of arthritic mouse (Figure 9). Serum starvation method was used to induce injury in the form of apoptosis in FLS. Therefore, FLS were serum starved for 72 h followed by the addition of IRDye 800 labeled C2-Crry as mentioned in the Methods so that it can be detected on apoptotic FLS. Binding of IRDye 800 labeled C2-Crry to apoptotic FLS was observed using a Zeiss Axio Observer 5 epifluorescent microscope (Figure 9B). Apoptotic cells with bound C2-Crry appeared green (Figure 9B) compared with non-apoptotic control FLS (Figure 9A). Apoptosis in FLS was separately confirmed using an agarose gel by visualizing a DNA ladder of 1 Kb, characteristic of apoptotic cells as shown in Supplementary Figure 3. Serum starved FLS for 6 h were used, as a negative and positive controls, and stained with Caspase 3/7 to show specific apoptosis of FLS induced by serum starvation under UV light (Figures 9C,D). These serum starved FLS data show that C2-Crry specifically targeted apoptotic, but not healthy FLS.

### *In vivo* Targeting and Detection of IRDye 800 Labeled Anti-CII Antibodies and C2-Crry in the Joints of Arthritis Mice

**FIGURE 10:**
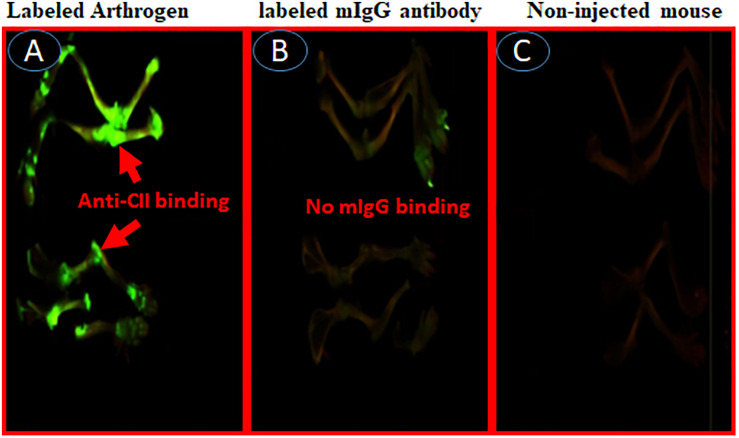
*In vivo* imaging of binding of IRDye 800 labeled Arthrogen to the cartilage or joints in mice. Showing specific localization of IRDye 800 labeled-Arthrogen (green color), at day 15, to the cartilage in the joints of mice. All mice were injected i.v. with IRDye 800 labeled Arthrogen and sacrificed after a long wash period of 15 days **(A)** Labeled Arthrogen (green color) localized and specifically bound to the joints. **(B)** Labeled murine IgG control (no green color) s.c. injected mice showing no specific binding or localization to the joints. **(C)** Non-injected mice as control (no green color) in the joints. Scanning/and or imaging using Li-COR Odyssey. No scale was added when the images were taken and images of the entire limb(s) has been shown. These experiments were performed three times using three mice in with each treatment with excellent reproducibility.

To examine whether IRDye 800 labeled C2-Crry specifically targeted joints in mice with arthritis, two experiments were performed. In the first experiment, we injected WT C57BL/6 mice with IRDye 800 labeled (green color) anti-CII Abs, or with control IRDye 800 labeled mouse IgG in mice (Figure 10). At day 15, all mice were sacrificed after a long wash period of 3 days. Only labeled anti-CII Abs, but not murine labeled IgG, localized in the joints (Figures 10A,B). As expected no green color was detected in the joints of non-injected mice (Figure 10C). In separate studies, mice injected with 1 × PBS, appears identical to the non-injected mice (data not shown) and we have published it (61). No non-specific binding of IRD800 labeled anti-CII Ab was seen in other organs such as kidney, liver and lung which is consistent with our previous publication therefore no images were taken (61). These data show that IRDye 800 labeled antibodies or proteins can be detected in injured joints.

**FIGURE 11:**
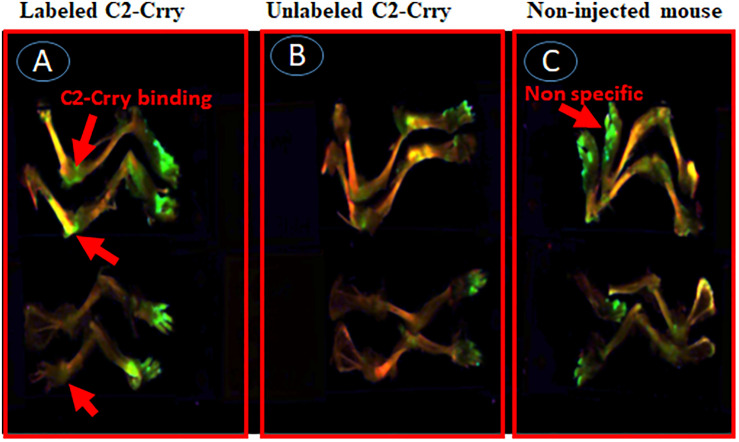
*In vivo* scanning using Li-COR Odyssey of binding of IRDye 800 labeled C2-Crry binding to the cartilage in mice with CAIA. IRDye 800 labeled (Green color) C2-Crry localized and bound to the joints in arthritic mice with arthritis at day 15. Suboptimal disease (injury) was induced in mice joints by injecting Arthrogen and LPS as mentioned in Methods. All mice were sacrificed 3 days after injection (i.v.), due to short half-life, with labeled or unlabeled C2-Crry followed by imaging. **(A)** Labeled C2-Crry (green color shown by red arrow) bound to the joints of arthritic mice with CDA score 6. **(B)** Unlabeled C2-Crry not detected in the joints of arthritic mice with CDA 7. **(C)** Non-injected control mice show no binding as expected in the joints of normal mice with CDA 0. Non-specific fluorescence (shown by red arrow) has been seen on paws of all mice due to excretion of the dye in urine of labeled C2-Crry mice for all mice were kept in the same cage for the purpose of blinding. No scale was added when the images were taken and images of the entire limb(s) has been shown. These experiments were done two times using three mice each time.

In the second experiment, a suboptimal disease was induced in WT C57BL6 mice by injecting unlabeled Arthrogen (Figure 11). At day 15, these mice were injected with IRDye 800 labeled C2-Crry or unlabeled C2-Crry and sacrificed after a wash period of 3 days, i.e., at day 18. Only IRDye 800 labeled C2-Crry bound to the knee joints, (Figure 11A). Although the binding of IRDye 800 labeled C2-Crry was not as robust when compared with IRDye 800 labeled anti-CII Abs, which might be due to the short half-life of C2-Crry, no distinct green color is visible in the joint of mice injected with non-labeled C2-Crry (Figure 11B). Similarly as expected no green color was detected in the joints of non-injected mice (Figure 11C). Of-note, some labeled C2-Crry was excreted rapidly in the urine of mice, which resulted in a non-specific binding on the paws (green color) of all mice even in non-injected mice for all these mice were kept in one cage due to blinding (Figures 11A–C). No non-specific binding of IRD800 labeled C2-Crry was seen in any other organs such as kidney, liver and lungs therefore no images were taken. These *in vivo* IRDye 800 C2-Crry labeling images show that C2-Crry can specifically target injured joints in mice.

## Discussion

In this study, we first showed that anti-C2 IgM NAb specifically recognized surface epitope(s) on apoptotic thymocytes. Second, C2-IgM NAb was shown to enhance suboptimal injury in CAIA, a complement-dependent mouse model of inflammatory arthritis. Third, we recombinantly linked C2scFv with the complement inhibitory protein Crry and then generated and purified a fusion protein, C2-Crry, in which the C2 sequences retained binding capacity to PL on apoptotic or injured cells. C2-Crry was also shown to attenuate arthritis in CIA, a separate complement dependent mouse model of arthritis. Finally, IRDye 800 labeled C2-Crry specifically bound *in vitro* to injured sFLS derived from an arthritic mouse, and *in vivo* imaging data showed that C2-Crry bound to the injured arthritic knee joints in mice. Overall, these data suggest that C2-Crry can be clinically useful for the treatment of arthritis or other autoimmune diseases involving apoptosis.

Notably, over one million cells die every second in the human body (62), and cell death can also occur due to infections such as viruses and bacteria as a primitive way of limiting their replication and systemic spread (63). Apoptosis pathways have also been linked to tolerance and immunity (64). The main purpose of apoptosis is to dispose of unwanted cells in a physiological controlled manner (65, 66) and in doing so cells expose eat me signals, e.g., phosphatidylserine, on their surface. We have shown previously that but C2-IgM binds to phosphatidylserine (48), although the actual epitope (s) to which C2 binds on apoptotic cells still unknown; however, it was speculated that C2 might bind to glycerol/fatty acid interface (48). The membrane contents of the cells undergoing apoptosis are well preserved and can still interact with the surrounding microenvironment, including NAbs already present in the blood.

Natural antibodies, in contrast to adaptive immune response generated antibodies, are present in mammals without previous externally generated antigen exposure, vaccination or infection. In contrast to antigen-induced antibodies, which are mainly IgG and mono-reactive, many NAbs are IgM and polyreactive. NAbs are self-reactive IgM Abs and mainly secreted by CD5 + B1 cells even in germ free environments and constitute up to 80% of the systemic IgM levels (49, 67, 68). Because of the NAb ability to bind self-antigens, they may serve as templates for some of the high-affinity autoantibodies that emerge in patients with autoimmune disease, particularly those associated with a significant expansion of CD5 + B cells (69–71). Thus, chromic activation of the immune system can lead to expansion of naturally present auto poly-reactive clones of NAbs. Furthermore, in genetically predisposed individuals, this can lead to the development of autoimmune diseases such as arthritis and lupus.

Apoptotic and necrotic cell death, dependent on the CS, also occurs due to tissue reperfusion after ischemia (72). C2 NAb neoepitopes have also been shown to be exposed in post-ischemic mouse models of intestine and brain injury (47, 48). Previous studies have shown that other self-reactive IgM NAbs recognize non-muscle myosin and catalyze intestinal (73), myocardial (74), and skeletal muscle (75) ischemia reperfusion injury in Rag1 deficient mice; an identical antibody specificity is found in human serum (76). In another model of recognition of annexin-IV by a pathogenic Nab, by transplanting hearts from WT donor mice into antibody deficient mice reconstituted with specific self-reactive IgM mAbs neoepitope, Annexin-IV expressed on post-transplant heart was shown to be the key mechanisms for IgM recognition of neoepitopes in graft injury (49). Similarly, anti-Annexin scFV linked to Crry (a.k.a. B4-Crry) not only blocked graft IgM binding and complement activation but also reduced graft inflammation and injury (49). In that study, whether B4-Crry actually bound to the apoptotic cells or not was not clear. Our data clearly show that IRDye 800 labeled C2-Crry bound to the FLS, *in vitro*, undergoing apoptosis due to serums starvation (Figure 9B) and also, *in vivo*, bound to the knee joints of arthritic mice (Figure 11A). Apoptosis in serum starved FLS was also separately confirmed using annexin V plus propidium iodide by flow cytometry analysis (data not shown). All of above mentioned studies provided the fundamental concept that IgM NAbs can cause injury and targeted delivery of scFv-linked complement inhibitory proteins can reduce injury in ischemia reperfusion related injury mouse models. These results are consistent with our current study in which C2 IgM mAb induced inflammation but scFV-linked Crry with C2 reduced inflammation, i.e., arthritis probably by targeting to apoptotic sFLS in the knee joints.

In this study, we have targeted pathologically important epitopes in the form of PL generated during injury or inflammation in the synovium. FLS are the predominant cell type present in the pannus (multilayered synovium) of inflamed joints in RA. In addition, other cell types such as T cells, B cells, adipocytes, monocyte, neutrophil and macrophage are also present. Furthermore, the cartilage surface gets damaged during inflammation due to the direct binding of anti-CII antibodies (5, 77). Using a conjugate of C2-Crry, we systemically delivered Crry to targeting the site of injury due to the specific neoepitopes for C2 IgM NAb expressed on the surface of synoviocytes in the synovium or on chondrocytes on the surface of cartilage. This is based on the imaging data that IRDye 800 labeled C2-Crry localized in the knee joints of arthritic mice (Figure 11A) compared with non-labeled C2-Crry (Figure 11B) indicating that C2-Crry bound to the injured synoviocytes or damaged cartilage surface. In addition, C2 IgM NAb alone enhances injury (Figure 2A) which normally leads to more C3 deposition as it is evident from mice with CIA, i.e., only PBS treatment have more C3 deposition compared with C2-Crry treated CIA mice (Supplementary Figure 2). One of the important questions is that whether C2-Crry will be effective when CS is compromised, e.g., in C3 deficient mice? We speculate that it will equally be effective because C2 might be binding to PL on apoptotic or injured cells independently from C3. Furthermore Crry possesses decay-accelerating activity for the CP but weak decay-accelerating activity for the AP C3 convertase so it might be equally effective in the complete absence of C3 because it can still inhibit CP C5 convertase although it has not been tested. We then asked the question what is the relevance of C2-Crry for its clinical use as a medicine in RA patients because Crry is not present in humans? In this study we have provided the proof-of-concept, *in vitro* and *in vivo*, that complement inhibitory protein such as Crry can be specifically delivered to the injured cells or injured joints using C2 scFv. In humans, CR1 is the functional orthologue of Crry, as it expresses cofactor and decay-accelerating function for both the CP and AP. Thus, the approach of delivering complement inhibitory protein, Crry or potentially using CR1, through C2 scFV as an apoptotic cell guide has also advantage over currently available therapeutic approaches which delivers complement inhibitory proteins systematically instead of direct targeted delivery to the injured tissue.

There are several limitations in our current study. One of the limitations is that we have not used C2 scFv alone to determine its cause and effect on the CAIA model. However, C2 scFv alone should not demonstrate a substantial effect on arthritis because it lacks the Fc domain and, therefore, will not activate complement. However, it may block certain neoepitopes and diminish joint damage in CIA. That remains to be evaluated. Furthermore C2-Crry has no known complement-dependent cytotoxicity. It does not contain any part of Fc which can bind to C1q. It is a single chain mAb and only contains variable regions. The second limitation is that we were unable to show through *in vivo* imaging a comparable amount of IRDye 800 labeled C2-Crry deposited in the joints of arthritic mice compared with labeled control anti-CII antibody (Figure 10A). There can be many reasons for this low deposition of C2-Crry in the joints. First, it might be related to the half-life of C2-Crry fusion protein, which was less than 48 h but more than 24 h. Second, IRDye 800 labeled C2-Crry clearance through urine, perhaps because of its small size allowing passage through the glomerular basement membrane, suggest it has less availability to apoptotic cells in the joints. Third, there might be less apoptotic cells in the joints due to the use of a suboptimal level of injury to dissect the protective effect of C2-Crry. Finally, based on our experience, apoptotic cells might be cleared rapidly from the synovium. In contrast, the reason, we do see more IRDye 800 labeled anti-CII deposited in the joints because it specifically binds to the collagen/chondrocytes on the surface of cartilage and causes injury. Some of the non-specific binding of IRDye labeled C2-Crry on the hair of paws of mice was noticed due to its excretion in the urine for all mice were kept in the same cage for the investigators were blinded to the treatment. Finally using and corroborating IRD800 labeled C2-Crry histology along with anti-C2-Crry immunohistochemistry might have yielded more valuable joint-specific binding information.

We also asked how the presence of NAbs and also the binding of C2 and C2-Crry have any clinical relevance to the treatment of RA. In early stages of apoptosis, nuclear chromatin condenses and DNA is digested into nucleosome-sized 180 base pair fragments (Supplementary Figure 3). Although there are not many reports related to the presence of apoptosis in RA synovium, it has been shown in 12 RA synovium and 4 OA synovium biopsies that apoptosis occurs and the primary site for apoptosis was the synovial sub lining (78). That study also showed that apoptosis can be induced in cultured sFLS with cytokines present in the inflamed joints of RA patients. It is consistent with our *in vitro* FLS data, in which apoptosis, was induced using serum starvation and then IRDye 800 labeled C2-Crry bound to these apoptotic FLS (Figure 9B). Interestingly, in the above mentioned study, synovial sub lining cells with fragmented DNA, i.e., apoptotic cells included macrophages and fibroblasts, but T cells in lymphoid aggregates, which expressed large amounts of bcl2, were spared (78). This study highlighted the fact that apoptosis specifically occur in the RA and OA synovium, but to what extend is not known. Furthermore, most of the rheumatology-related literature points out that apoptosis is a common feature of the RA synovium either because it occur naturally or it is induced by drugs which are currently being used for the treatment of RA. For example, Rituximab, anti-CD20 chimeric mouse/human mAb or CD40 fully humanized anti-CD20 mAb being used for the treatment of RA deplete B cells via apoptosis (79–81). In RA patients Fas and Fas-L have been detected in synovial cells and, also in activated mature T cells obtained at the time of arthroplasty (82) and these are highly susceptible to Fas-mediated apoptosis induced by anti-Fas mAb. Nonetheless, apoptosis pathways are defective in RA synovium and treatment with DMARD reduces Fas expression on the synovial tissue (82). In contrast, other studies have shown DMARD itself can initiate apoptosis both *in vitro* (83, 84) and *in vivo* (85). Another study support the concept that both Etanercept and Infliximab did induce apoptotic pathways in RA synovial tissue (86).

The exact relationship between pannus formation (synovial hyperplasia) and apoptosis in rheumatoid synovium is unknown. There might be an imbalance between cell proliferation and apoptosis in RA synovium that leads to pannus formation (87). Nonetheless apoptotic cells in the synovial lining can be a potential therapeutic target for fusion proteins like C2-Crry which specifically binds to PL. Even if apoptosis is not present then transient induction of apoptosis by drugs in the synovium or in the pannus could be a way to locally deliver C2-Crry in the RA synovium at very early or at very late stage of the disease.

We have previously shown that the LP of the CS plays an important role not only activating the AP but also directly playing an important role in inducing arthritis in mice (8, 88). But we do not know the precise interactions between NAbs, apoptosis and various LP components such as MBL or ficolins or collectins at very early stage in arthritis. We also do not know the extent of apoptosis in the peripheral blood or synovium or the presence of CD5 + B1 IgM NAbs producing cells in the synovium at very early stage disease in RA patients. Furthermore, whether rheumatoid factor (RF) IgM, which exists in RA, can act as a NAb is also not fully understood. RF are present in more than 70% of RA patients, and high titers are associated with severe disease (89). RF are also abundant in the RA synovium. Two types of RF exists, i.e., low affinity and high affinity RF. Low affinity RF are IgM NAbs, polyreactive, T cell independent and also produced by CD5 + B1 cells in normal subjects (89).

In sum, our study offers the potential for the development of new targeted drugs to inhibit complement activation in the joints at very early stage of RA that would be triggered by IgM Nabs as well as disease specific IgG antibodies. Other fusion proteins such as C2-MAp44 or C2-sMAP can be designed to directly deliver a LP inhibitors MAp44 or sMAP to the joints, or C2-Factor H to deliver an AP, could also be created and tested in studies going forward.

## Data Availability Statement

All datasets presented in this study are included in the article/Supplementary Material.

## Ethics Statement

The studies involving human participants were reviewed and approved by IRB University of Colorado. The patients/participants provided their written informed consent to participate in this study. The animal study was reviewed and approved by IACUC University of Colorado.

## Author Contributions

VH conceived the idea for making fusion protein, C2-Crry, supervised the project and reviewed this manuscript in-depth. NB proposed the idea of testing of C2-Crry *in vivo*, planned and executed all *in vivo* CIA and CAIA experiments, designed labeling of Arthrogen and C2-Crry with IRDy800, analyzed all data and wrote the first and revised drafts of the manuscript. ST originally designed, constructed and purified fusion protein, C2-Crry-6Histag. LK cloned NAb C2, and purified already clones fusion protein, C2-Crry and checked the quality of all NAbs used and also C2-Crry, respectively. LK also done the functional assays such as ELISA and FACS to show the specificity of C2 mAb binding to phospholipids. NH, JR, and GM assisted in all *in vivo* CAIA and CIA as well as IHC studies. RS, VV, GW, and DS assisted in planning labeling of Arthrogen IRDy800 and standardization of *in vivo* injection planning, and strategy for determining the *in vivo* half-life of C2-Crry and imaging joint of mice. All authors contributed to the article and approved the submitted version.

## Conflict of Interest

NB: C2-MAp44 fusion protein licensed to AdMIRx. ST, LK, and VH: AdMIRx. The remaining authors declare that the research was conducted in the absence of any commercial or financial relationships that could be construed as a potential conflict of interest.

## Supplementary Material

Supplementary Figure 1 Photographs of C2-IgM NAb enhanced inflammation in CAIA mice at day 3 and at day 10. **(A)** No visible inflammation (shown in circle by red arrow) on the forepaws of mice treated with control F-632 IgM NAb. **(B)** Visible inflammation (shown in circle by red arrow) on forepaws of mice treated with anti-C2-IgM. **(C)** Inflammation (shown in circle by red arrow) on forepaws of mice treated with 1 × PBS (control). **(D)** Inflammation (shown in circle by red arrow) on forepaws of mice treated with control F-632 IgM. **(E)** Substantial inflammation (shown in circle by red arrow) on forepaws of mice treated with anti-C2-IgM. All photographs were taken by simple polaroid camera under normal light.

Supplementary Figure 2 Representative histopathologic images of inflammation, C3 deposition and macrophage staining from the knee joints of CIA mice injected i.p. four times with PBS or C2-Crry fusion protein. At day 35, all joints were fixed with 10% neutral buffered formalin, decalcified, paraffin embedded, and then sectioned at a thickness of 5 μm. The left hand side two panels top and bottom **(A,B)** show inflammation, i.e., staining with toluidine-blue (blue color) from the knee joints of CIA mice treated with PBS (left top panel) or with C2-Crry (right bottom panel). The middle set of two panels top and bottom **(C,D)** show C3 deposition (brown color) staining from the knee joints of CIA mice treated with PBS (Center top panel) or with C2-Crry (Center bottom panel). The right hand side two panels top and bottom **(E,F)** show macrophage F4/80 (red color) staining of knee joint of CIA mice treated with PBS (right top panel) or with C2-Crry (right bottom panel). Areas of synovium (S-black arrow), cartilage (C-black arrow), bone (B-black arrow), and meniscus (M-black arrow) are identified. The sections for inflammation (blue color) were photographed under the 20x objective while sections of C3 deposition and macrophage IHC staining were photographed under the 10x objective. Scale bars have shown in the red at the right bottom knee joint equal 0.05 mm (50 μm) for 20x and 0.01 mm (100 μm) for 10x objectives, respectively.

Supplementary Figure 3 Detection of DNA apoptotic ladder in serum starved FLS. FLS with no serum starvation (lane 1; no DNA ladder) or FLS with serum starved for 3 days to induce apoptosis (lane 2; DNA ladder present) or DNA ladder marker of 1 Kb (lane 3). Genomic DNA from FLS grown in the presence or absence of serum was isolates using QIAGEN DNA kit. 4 μg of DNA from each sample was electrophoresed in 1 × TAE buffer, 90 volts for 1 h using 1.2% agarose gel containing Ethidium bromide followed by visualization under UV light.

## Abbreviations

AJM: all joint mean
AP: alternative pathway
CAIA: collagen antibody-induced arthritis
CDA: clinical disease activity
CIA: Collagen-induced arthritis
CII: Bovine type II collagen
CP: classical pathway
Crry: Mouse complement receptor-related y
CS: complement system
C2-scFv, C2scFv-Crry: C2-single-chain variable fragment
FLS: fibroblast-like synoviocyte
IgM: Immunoglobulin M
LP: lectin pathway
LPS: lipopolysacharide
mAb: monoclonal antibody
Nabs: natural antibodies
WT: wild type.
