# Supplementary figures and images for: C2 IgM Natural Antibody Enhances Inflammation and Its Use in the Recombinant Single Chain Antibody-Fused Complement Inhibitor C2-Crry to Target Therapeutics to Joints Attenuates Arthritis in Mice
Source: Front Immunol. 2020 Oct 16;11:575154. doi: 10.3389/fimmu.2020.575154 (PMC7596757; doi:10.3389/fimmu.2020.575154)

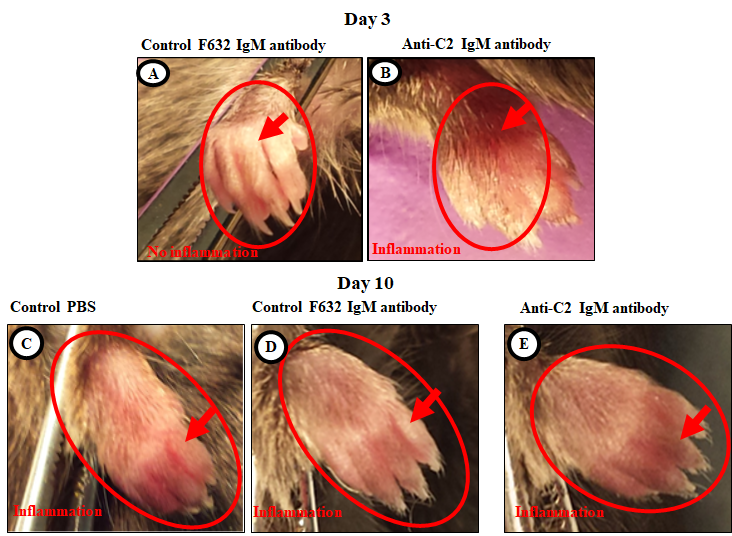

Supplement: Supplementary Figure 1 — Photographs of C2-IgM NAb enhanced inflammation in CAIA mice at day 3 and at day 10. (A) No visible inflammation (shown in circle by red arrow) on the forepaws of mice treated with control F-632 IgM NAb. (B) Visible inflammation (shown in circle by red arrow) on forepaws of mice treated with anti-C2-IgM. (C) Inflammation (shown in circle by red arrow) on forepaws of mice treated with 1 × PBS (control). (D) Inflammation (shown in circle by red arrow) on forepaws of mice treated with control F-632 IgM. (E) Substantial inflammation (shown in circle by red arrow) on forepaws of mice treated with anti-C2-IgM. All photographs were taken by simple polaroid camera under normal light. [file Image_1.TIF]

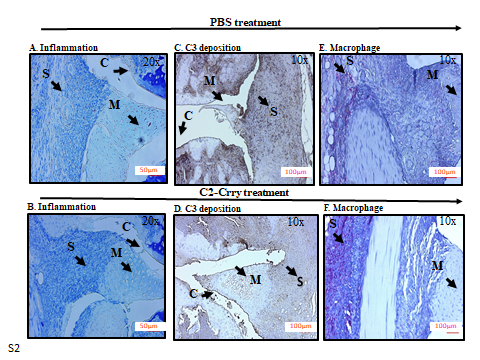

Supplement: Supplementary Figure 2 — Representative histopathologic images of inflammation, C3 deposition and macrophage staining from the knee joints of CIA mice injected i.p. four times with PBS or C2-Crry fusion protein. At day 35, all joints were fixed with 10% neutral buffered formalin, decalcified, paraffin embedded, and then sectioned at a thickness of 5 μm. The left hand side two panels top and bottom (A,B) show inflammation, i.e., staining with toluidine-blue (blue color) from the knee joints of CIA mice treated with PBS (left top panel) or with C2-Crry (right bottom panel). The middle set of two panels top and bottom (C,D) show C3 deposition (brown color) staining from the knee joints of CIA mice treated with PBS (Center top panel) or with C2-Crry (Center bottom panel). The right hand side two panels top and bottom (E,F) show macrophage F4/80 (red color) staining of knee joint of CIA mice treated with PBS (right top panel) or with C2-Crry (right bottom panel). Areas of synovium (S-black arrow), cartilage (C-black arrow), bone (B-black arrow), and meniscus (M-black arrow) are identified. The sections for inflammation (blue color) were photographed under the 20x objective while sections of C3 deposition and macrophage IHC staining were photographed under the 10x objective. Scale bars have shown in the red at the right bottom knee joint equal 0.05 mm (50 μm) for 20x and 0.01 mm (100 μm) for 10x objectives, respectively. [file Image_2.TIF]

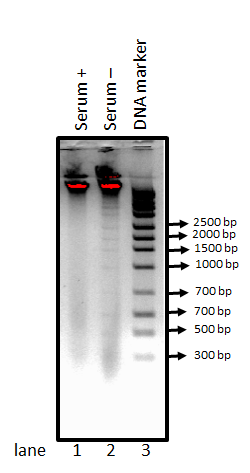

Supplement: Supplementary Figure 3 — Detection of DNA apoptotic ladder in serum starved FLS. FLS with no serum starvation (lane 1; no DNA ladder) or FLS with serum starved for 3 days to induce apoptosis (lane 2; DNA ladder present) or DNA ladder marker of 1 Kb (lane 3). Genomic DNA from FLS grown in the presence or absence of serum was isolates using QIAGEN DNA kit. 4 μg of DNA from each sample was electrophoresed in 1 × TAE buffer, 90 volts for 1 h using 1.2% agarose gel containing Ethidium bromide followed by visualization under UV light. [file Image_3.TIF]
